# The Outdoor Care Retreat: Key Insights for Navigating Biophilic Design Innovation in Healthcare Environments

**DOI:** 10.1177/19375867251365849

**Published:** 2025-09-03

**Authors:** Eli Kindervaag, Åshild Lappegard Hauge, Svein Åge Kjøs Johnsen

**Affiliations:** 1 University of Inland Norway, Lillehammer, Norway; 2 University of Oslo/Inland Norway University of Applied Sciences, 6305 University of Oslo, Oslo, Norway

**Keywords:** biophilic design, biophilia, healthcare design, healthcare environments, healthcare innovation, public sector innovation

## Abstract

**Aim:**

The present case study explores insights related to navigating biophilic design innovation in public healthcare using the Outdoor Care Retreat at Oslo University Hospital and the Hospital of Southern Norway as a case.

**Background:**

Available research from environmental psychology proposes an association between access to biophilic design, well-being, health, and hospitalization satisfaction. Still, integrating this research into public healthcare is challenging and there is a need for research that builds on innovative examples of biophilic design integration in this context.

**Methods:**

Study findings are based on eight individual interviews with people involved in project initiation and development of the Outdoor Care Retreat, in addition to online public records and other digital documents related to the case study. The qualitative materials were analyzed according to reflexive thematic analysis and categorized into themes.

**Results:**

Findings illustrate how moving beyond conventional healthcare practices can support the implementation of biophilic design. Specifically, the findings suggest that biophilic design in healthcare benefits from an appealing story, individual contributions, and an organizational and environmental context that challenges common conventions. Drawing from research from public healthcare innovation and biophilic design, the study outlines key insights for implementation.

**Conclusions:**

This study contributes to bridging the gap between theory and practice in a public healthcare context. It offers key insights for implementing biophilic design in healthcare environments, with the potential to ensure valuable improvements in the hospitalization experience for patients and their families in future developments of public healthcare facilities.

## Introduction

A substantial body of research and literature in environmental psychology and related fields has identified a positive association between access to nature and biophilic design and well-being, health outcomes, and hospitalization satisfaction (Guidolin et al., 2024; Hung & Chang, 2021; Kellert, 2018). Still, findings from this research field have generally had few substantial long-term implications for healthcare design (Altringer, 2010). To ensure patient access to environments that promote and optimize well-being and increase hospitalization success, there is a need for research that can build on innovative examples of biophilic healthcare designs and how these were initiated and developed.

*To ensure patient access to environments that promote and optimize well-being and increase hospitalization success, there is a need for research that can build on innovative examples of biophilic healthcare designs and how these were initiated and developed*.

The present study investigates public healthcare innovation, specifically exploring insights related to initiation and development of a biophilic design alternative to healthcare environments by using the Outdoor Care Retreat project at Oslo University Hospital and the Hospital of Southern Norway as a case. Drawing on perspectives from environmental psychology, public innovation, and healthcare innovation research, the study aims to provide insights from the initiation and development of the Outdoor Care Retreat to inspire stakeholders to utilize innovation strategies to bridge the gap between research and practice to ensure patient access to biophilic design and the associated benefits for health and well-being. To our knowledge, no previous studies have applied frameworks from innovation research to explore the development of healthcare designs based on principles of biophilic design.

### Biophilic Healthcare Design

The Outdoor Care Retreat provides hospitalized patients and their families with an opportunity to engage with nature during short or extended hospital stays and its architectural features may serve as an example of biophilic design. Kellert's principles of biophilic design emphasize the innate human tendency to feel emotional affinity with other living organisms, in line with the biophilia hypothesis (Kellert & Wilson, 1993; Wilson, 1984). His approach organizes biophilic design into two dimensions: the organic or naturalistic dimension, which incorporates natural forms, materials, and sensory experiences; and the place-based or vernacular dimension, which fosters connection to local ecology, culture, and geography. These are expressed through six key elements: environmental features, natural shapes and forms, natural patterns and processes, light and space, place-based relationships, and evolved human-nature relationships. Together, these principles aim to create environments that support human health, well-being, and productivity by integrating meaningful, multisensory experiences of nature into design (Kellert, 2018). Decades of research in environmental psychology have demonstrated an important association between spending time in natural settings and increased mental and physical well-being (Elsadek et al., 2019; Ewert & Chang, 2018; Gaekwad et al., 2023; Lee et al., 2009; Ulrich et al., 2010). By extension, spending time in nature and biophilic environments has been hypothesized and shown to have certain healing properties, both on its own and when used as part of conventional treatment and therapy (Gaekwad et al., 2023; Tekin et al., 2023; Ulrich et al., 2010). Existing research on the Outdoor Care Retreat largely supports previous research on human-environment interactions and suggests beneficial outcomes for patients, their families, and staff when used as part of therapy and as a place to spend time during challenging hospital stays (Hauge et al., 2023; Kindervaag et al., 2024). In a qualitative study of children's experience of the Outdoor Care Retreat, it was found that the space activates different behavior modes than the conventional hospital spaces and that children feel less restricted and more free to express themselves and a wider range of emotions in the Outdoor Care Retreat environment (Kindervaag et al., 2024). Additional research has found that the Outdoor Care Retreat contributes to therapeutic flow that benefits both children and therapists (Hauge et al., 2023). While the benefits of the Outdoor Care Retreat on end-users are documented, less is known about how such innovations can be developed and implemented in public healthcare. Barriers of implementing biophilic design in healthcare environments can include cost constraints, space- and structural limitations, building codes and standards, as well as consideration of cultural context and practices (Dion et al., 2023; Tekin et al., 2023; Tomasso et al., 2021). The Outdoor Care Retreat is unique and innovative as it minimizes costs and space limitations by being a small cabin located outside the main hospital buildings, while still ensuring access to the proposed benefits of spending time in biophilic environments. Thus, an innovation perspective on the process of developing this space is seen as a valuable approach to better understand how innovative biophilic design can be implemented in public healthcare.

### Healthcare Design and Public Innovation

Public healthcare must seek continuous development to reduce cost and utilize resources in an effective way, while simultaneously ensuring safe and high-quality patient care that aligns with best available evidence (Kennel & Lowndes, 2023). It takes place in shared environments (e.g., hospitals) and in a context of government and governance where it is necessary to align with certain requirements regarding public ethos, while adhering to strict policies and intense regulations to ensure safe and high-quality patient care (Kennel & Lowndes, 2023). Innovation in public healthcare is important to ensure continued quality improvement of patient care and to optimize patient safety and well-being (Flessa & Huebner, 2021; Kennel & Lowndes, 2023; Raine et al., 2016).

Innovation in this field has mostly been concerned with the processes of developing and implementing novel therapies, drugs, and devices, with less attention given to how innovation can be used to ensure more user-centered design (Lyon et al., 2020). Still, efforts have been made, and design has become increasingly central in public innovation, especially in terms of service design, co-design, co-creation, design thinking and participatory design, with increased awareness and consideration of user needs in healthcare design and services (Altman et al., 2018; Greenhalgh et al., 2016; Sanders et al., 2008).

One approach is Human-Centered Design, a field concerned with using quantitative and qualitative methods to understand contextual barriers and needs (Lyon et al., 2020). Human-Centered Design methods usually include performing a mapping of contextual factors specific to the situation, by using questionnaires, interviews, or workshops with intended users to suggest context-specific user considerations, wants, and needs. A second approach is Evidence-Based Design (Ulrich et al., 2010). Like Human-Centered Design, Evidence-Based Design aims to ensure a user-centered approach to design but favors the use of existing scientific evidence on the association between health and the built environment. These approaches are valuable routes to ensuring healthcare environments that align with user needs and satisfaction but do not necessarily provide a broad framework for understanding how innovative healthcare designs can be implemented. A related, but novel, perspective on implementation of biophilic healthcare design is to look at key elements of public innovation more broadly.

De Vries et al. (2016) suggest that public innovation can be explored by analyzing the organizational, cultural, and environmental context of the innovation, in addition to characteristics of people who innovate. At organizational level, the innovation must be compatible with the values and cultural norms of the organization to ensure success, as organizational and cultural resistance has been observed as a barrier for innovation (Sarooghi et al., 2015). Additionally, innovation requires an organizational culture that supports innovative ideas and personalities, and can benefit from transformational leadership where leaders inspire admiration, loyalty, and respect by promoting a sense of a collective goal. Collaboration between team members with diverse professional backgrounds can similarly facilitate innovation and innovation in healthcare (Kennel et al., 2017; Kennel & Lowndes, 2023). For instance, a study by Sonke and colleagues (Sonke et al., 2017) found that bringing professional artists into an interprofessional care team was considered valuable to nurses in facilitating quality improvements for patients and staff by providing a holistic approach to healthcare service.

Collaborative strategies for innovation can facilitate exchange of ideas, knowledge, and competence that can inspire mutual learning and allow a broader understanding of the challenge at hand and the optimal solutions to solve it (Torfing, 2018). The innovation literature has also given considerate attention to individual abilities and traits for innovation (De Haro & Vena, 2025; Thornhill-Miller & Lubart, 2019). Existing research suggests that innovative individuals have a predisposition for divergent and convergent thinking, mental flexibility, associative thinking, and knowledge (Sternberg & Lubart, 1999; Zhang & Sternberg, 2011). Additionally, these individuals are more open to experiences, willing to take risks, have a high tolerance for ambiguity, and high intrinsic motivation (Thornhill-Miller & Lubart, 2019; see also Amabile, 1988; Sternberg & Lubart, 1999). These traits and abilities have all been associated with innovative behaviors and innovation success.

### Aim and Research Question

This study explores key insights for developing innovative healthcare environments in public healthcare using the Outdoor Care Retreat project as a case. The study seeks to contribute to research in public healthcare innovation and environmental psychology by generating new knowledge based on the following research question:

RQ: What insights can the Outdoor Care Retreat project provide about public innovation and development of biophilic design in healthcare environments?

## Methodology

### The Outdoor Care Retreat

**Figure 1.**
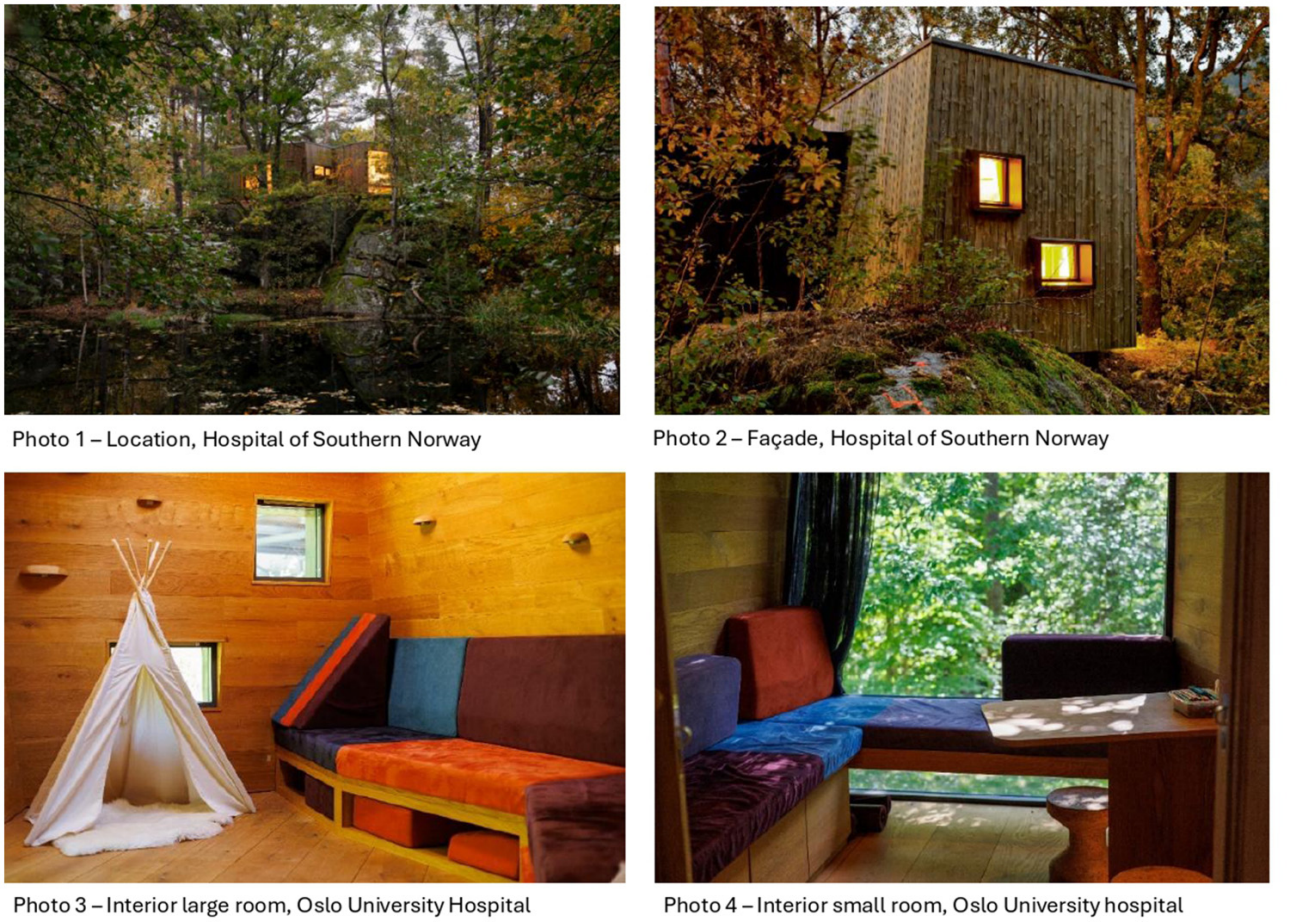
The outdoor care retreat.

The Outdoor Care Retreat is a small wooden cabin located in natural surroundings near the conventional hospital buildings. It has several distinct qualities, perhaps most evident is that it is a wooden cabin in a natural environment with noticeable design elements that make it easy to distinguish from more conventional hospital design (Figure 1, photos 1-2). At both locations, the view from inside the cabin faces natural landscapes, and the interior is clad with wood (Figure 1, photos 3-4). The building consists of two main rooms: one larger room with open space and one smaller room with a table. The layout of the rooms was designed in accordance with architecture in human scale, and the human preference to both move about freely while ensuring privacy (Gehl, 2011; Tekin & Urbano Gutiérrez, 2023). The interior consists of built-in wooden benches with pillows that can be used for play or relaxation. Today, the Outdoor Care Retreat is mainly used by hospitalized children with healthcare professionals in a therapeutic setting, and as a place for patients to spend time with family members outside the conventional hospital buildings. The Outdoor Care Retreat project was initiated and developed by frontline employees at the Department for Child and Adolescent Mental Health in Hospitals at Oslo University Hospital in collaboration with the parent of a long-term hospitalized child and an architecture firm between 2015 and 2018. The first two Outdoor Care Retreats opened in 2018 and the foundation is planning for Outdoor Care Retreats at other hospitals in the future (Lindheim et al., 2020).

*The Outdoor Care Retreat is a small wooden cabin located in natural surroundings near the conventional hospital buildings*.

### Research Design

To understand key insights for innovation inbiophilic healthcare design, we needed a qualitative and open research design, sensitive to contextual influence. The data material consisted of two data collections: 1) Individual interviews were carried out with eight stakeholders involved in the process of developing, designing, and implementing the Outdoor Care Retreat, and 2) online public records from the Agency of Planning and Building Services in Oslo municipality and other digital documents related to the case study. We chose reflexive thematic analysis as a guide for the methodological approach (Braun & Clarke, 2021). There are different variations of this approach, and we adhere to a critical reflexive thematic analysis, exploring implicit meanings in the data material based on a constructionist epistemology. This means that we recognize that our analysis is deductive, shaped by our interest in exploring insights related to public innovation (De Vries et al., 2016), and our understanding of the healing influence of nature (Gaekwad et al., 2023). Within a constructionist approach, the results are seen as socially constructed and generated in cooperation between the informant and the researcher. Thus, the background of the researchers and the reflexivity on this background becomes central (Braun & Clarke, 2021; Olmos-Vega et al., 2023).

### Reflexivity

Braun and Clarke (2021) highlight the researcher's active role in knowledge production as part of reflexive thematic analysis, and subjectivity is valued. Aligned with how researchers should reflect on how their background influences the interpretation of data and production of knowledge, it is considered relevant for the present study that the authors are familiar with environmental psychology and research-based design, as well as the role of nature and physical environments for well-being and health. All authors have previously published research on the Outdoor Care Retreat. Still, none of the authors have any financial interests in the case study, allowing for an impartial viewpoint on the subject matter. However, the desire for positive outcomes and to promote the healing benefits of nature might influence how the results are presented. On a positive note, our expertise in the topic could lead to an examination of details in the data that researchers from different backgrounds might overlook.

### Participants and Materials

Participants included the founder of the Outdoor Care Retreat Foundation, with a personal history as parent of a long-time hospitalized child; one frontline employee and the department leader from the Department for Child and Adolescent Mental Health in Hospitals at Oslo University Hospital; two architects responsible for designing the Outdoor Care Retreat; one sponsor; one project manager from the Hospital of Southern Norway; and one board member from the Outdoor Care Retreat Foundation. Participants are presented in Table 1.

**Table 1.** Participants (N = 8).

Participant	Characteristics	Project role
Founder of the Outdoor Care Retreat Foundation	Next of kin, founder	Key stakeholder
Frontline employee Oslo University Hospital	Clinical psychologist, founder	Key stakeholder
Department leader Oslo University Hospital	Medical doctor, professor, department leader	Key stakeholder
Architect	Responsible for Outdoor Care Retreat design	Contributor
Architect	Responsible for Outdoor Care retreat design	Contributor
Sponsor	Non-profit organization	Contributor
Project manager Hospital of Southern Norway	Responsible for implementation of an Outdoor Care Retreat at Hospital of Southern Norway	Contributor
Board member of The Outdoor Care Retreat Foundation	Involved with future development of the Outdoor Care Retreat Foundation	Contributor

**Figure 2.**
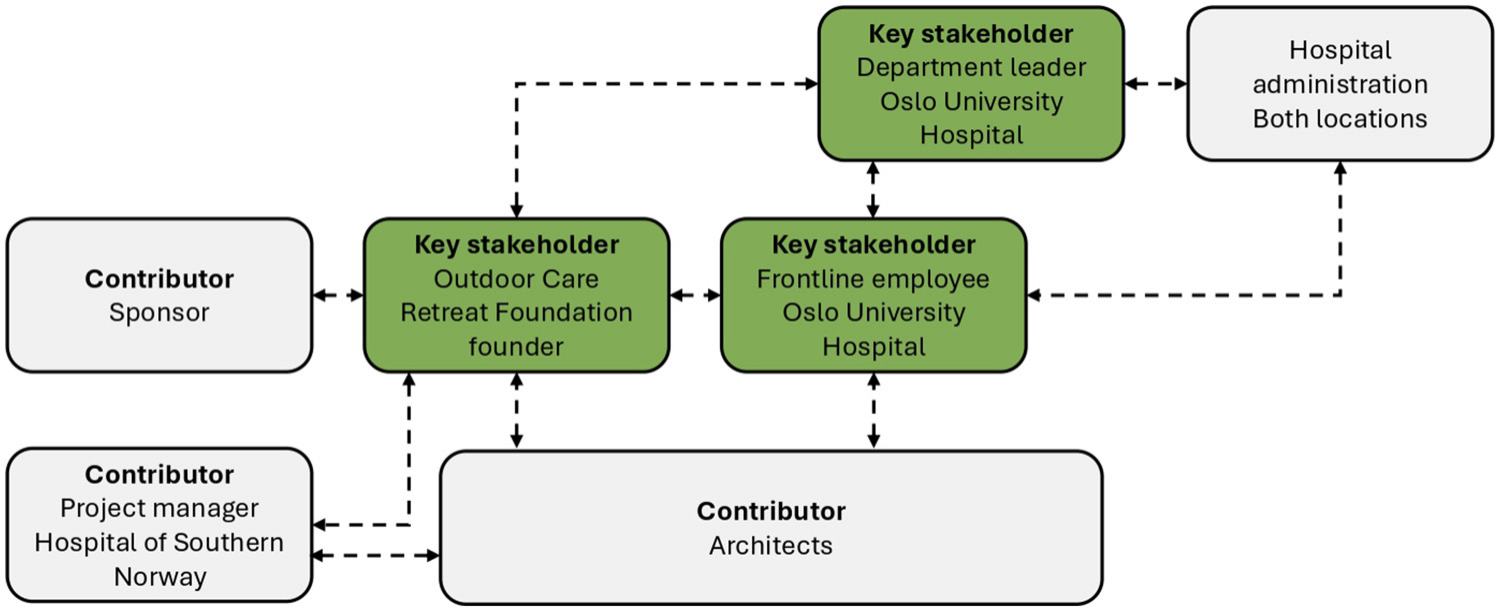
Overview collaboration and information flow.

**Figure 3.**
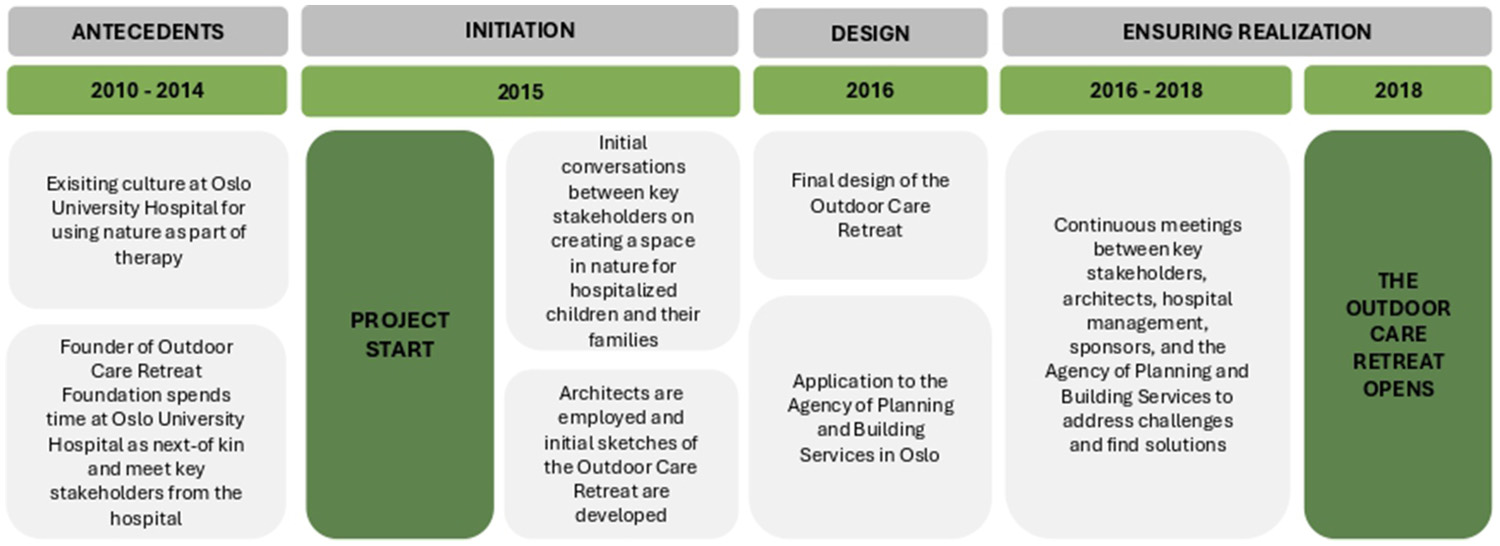
The innovation process.

Participants were recruited based on suggestions from the founder of the Outdoor Care Retreat Foundation and the frontline employee at Oslo University Hospital, both central to initiation and development of the Outdoor Care Retreat. Additional central stakeholders were identified and approached for participation in the study but unfortunately declined the offer to attend. This includes the department head at the time of project initiation and the project manager from Oslo University Hospital. In line with Malterud et al. (2016), we aimed for information power in selection of participants. Based on our aim of study, sample specificity, use of established theory, and a case study design, we concluded that the participant group represents sufficient information power. During analysis, participants were divided into key stakeholders and contributors, see Table 1. The collaboration and information flow between key stakeholders and collaborators are presented in Figure 2, and the innovation process from initiation to realization of the Outdoor Care Retreat is depicted in Figure 3.

In addition to interviews, online public records from the Agency of Planning and Building Services in Oslo municipality and other digital documents related to the study were obtained and included in the analysis. The documents are presented in Table 2.

**Table 2.** Documents Included in Document Analysis.

Document	Description
Sales proposal	Retrieved from sales proposal to St. Olav Hospital, Trondheim University Hospital. Includes background, vision, and additional facts about the Outdoor Care Retreat
Application letter	Retrieved from application to the Agency of Planning and Building Services in Oslo municipality. Includes background and vision for the Outdoor Care Retreat at Oslo University Hospital
Meeting minutes	Retrieved from application to the Agency of Planning and Building Services in Oslo municipality. Includes addressed challenges with solutions
Implementation plan	Retrieved from application to the Agency of Planning and Building Services in Oslo municipality. Includes detailed plan for implementation
Outdoor Care Retreat Foundation website	Background and vision for initiation and implementation of the Outdoor Care Retreat
Architecture firm website	Background and vision for the Outdoor Care Retreat Foundation
Newspaper article with interviews	Interviews with key stakeholders and architects on vision for the Outdoor Care Retreat

### Interviews

A semi-structured interview guide with open-ended questions was created. This allowed participants to reflect on experiences and discuss milestones, barriers, and successes from the development of the Outdoor Care Retreat. Interview topics are presented in Table 3.

**Table 3.** Semi-Structured Interview Guide.

Topic	Subtopic
Demographics	Age
	Gender
	Previous and/or current workplace/employer (during Outdoor Care retreat project development and current)
Process	Project role
	Collaboration with other project members and organizations (sponsors, hospital management)
	Organization of process
	Positive and negative experiences
	Success criteria and barriers
Design	Role in design process
	The design process
	Knowledge base for final design
	Intended users
Result	Opinions on the final result
	Success and areas of improvement

The first author conducted the interviews and had no previous experience with hospitalization and little knowledge about study participants prior to the interviews. The location of the interviews was decided by participants. The interview with the founder of the Outdoor Care Retreat Foundation and one of the architects was conducted at the Outdoor Care Retreat. One interview was conducted online. The interviews with employees at Oslo University Hospital were conducted in their work offices. The interview with one of the architects and the board member of the Outdoor Care Retreat Foundation was conducted in their own office space. The interviews were audio recorded and transcribed by the first author. On average, the interviews lasted 1 h 14 min (range: 39-114 min).

### Analysis

The qualitative material was analyzed according to reflexive thematic analysis (Braun & Clarke, 2021). This method of data analysis focuses on the researcher's active role in identifying patterns of meaning across a dataset and values reflexivity, meaning-making, and subjectivity in the process (Braun & Clarke, 2021). The analysis was guided by Braun and Clarke's (2021) six steps for reflexive thematic analysis: familiarization, coding, generating themes, reviewing themes, defining and naming themes, and write-up of results. These steps imply that all authors became familiar with the data material by reading interview transcripts and related digital documents. Following this, the data was coded, and initial themes were produced by the first author. The first round of analysis was semi-inductive to explore possible areas of interest that were not necessarily drawn from previous research. The codes and theme names were openly and repeatedly discussed and reviewed between the researchers, before the final themes were defined and named. The process of generating themes was latent, aiming at unpacking meanings which were not necessarily directly articulated by participants (Braun & Clarke, 2021).

### Ethical Considerations

The research followed proposed guidelines and was approved by Norwegian Agency for Shared Services in Education and Research. The authors found it important to inform participants of the possibility of being recognized in the written report based on widespread and non-anonymous media coverage of the Outdoor Care Retreat. The participants provided written consent and were simultaneously made aware that the topic for the present study was not of a sensitive nature.

## Results

### Beyond the System

**Figure 4.**
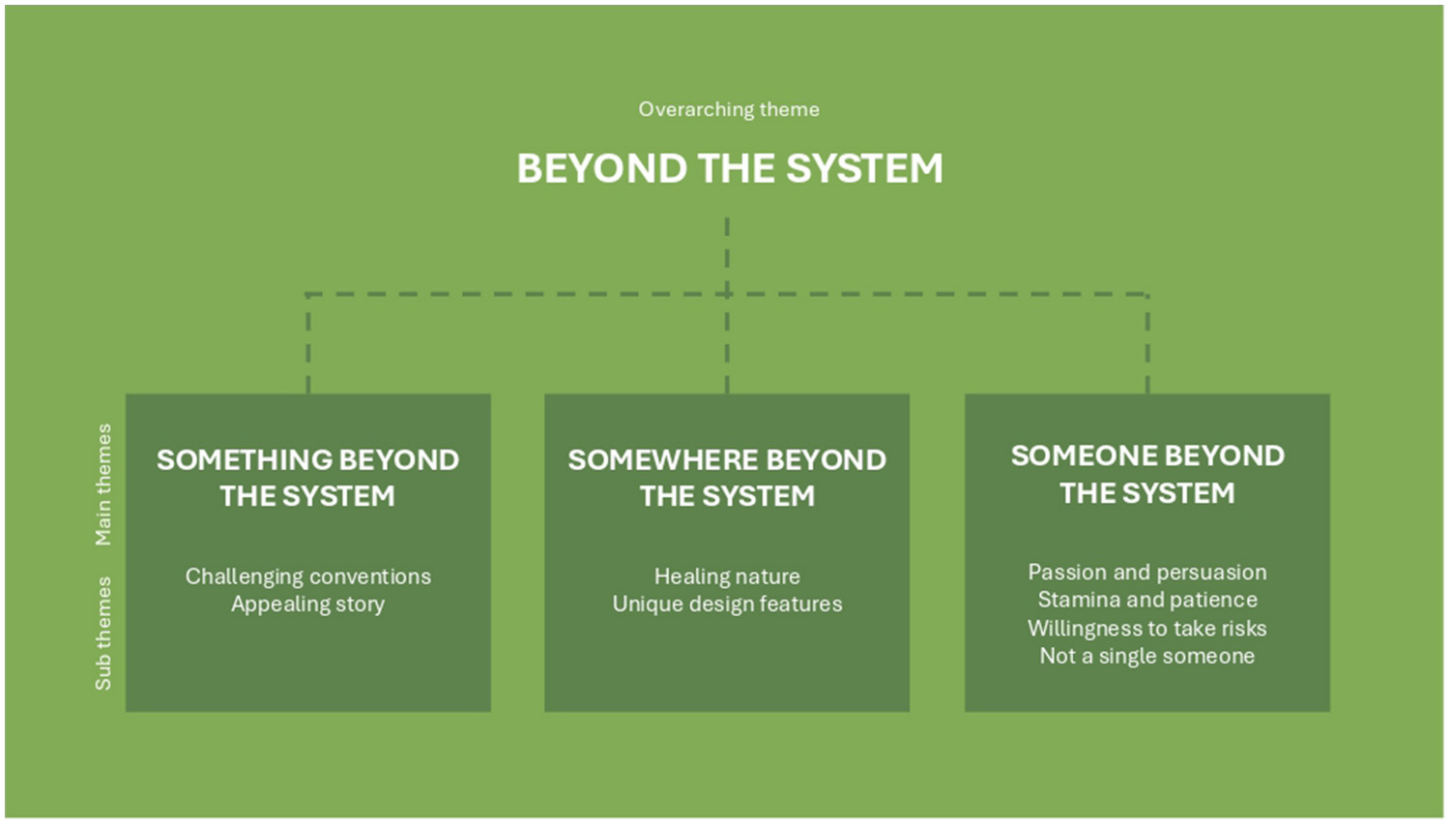
Overview overarching theme, main themes, and sub themes.

The results demonstrate how the process of developing the Outdoor Care Retreat was characterized by challenging common conventions in public healthcare at several levels and illustrate the influence of moving beyond the system for successful development of innovative biophilic design in public healthcare. To reflect this, an overarching theme (Braun & Clarke, 2021) “beyond the system” was created, with three sub-themes: 1) something beyond the system, 2) somewhere beyond the system, and 3) someone beyond the system. The themes are presented in Figure 4.

*The process of developing the Outdoor Care Retreat was characterized by challenging common conventions in public healthcare at several levels*.

### Something Beyond the System

The first main theme reflected how innovation success can be achieved by utilizing something beyond established conventions. This something could be anything that was experienced outside established frames of public healthcare. This theme was divided into (1) challenging conventions and (2) appealing story.

### Challenging Conventions

Most public healthcare products and services are financed by the public and managed by the hospital. The Outdoor Care Retreat project, however, was financed by external sponsors and offered as a gift to the hospital, thereby challenging these conventions. According to the project manager from the Hospital of Southern Norway, novel - and often existing - products, procedures, and services face brutal competition in hospital budgets and can act as a barrier to innovation. Thus, moving beyond traditional routes of funding in the hospital system contributed to project success.

Moreover, the Outdoor Care Retreat design challenges common conventions in hospital environments. The non-clinical and biophilic design is described as rare in a healthcare setting by participants. The stakeholders explained how the Outdoor Care Retreat is a physical manifestation of the specific departmental culture at Oslo University Hospital with their long-standing tradition of using nature as part of therapy and treatment. Overall, their approach includes encouraging divergent thinking and reflects an intent to challenge conventional perspectives on treatment and healthcare services. According to the stakeholders, this approach has received recognition by the hospital management and contributed to departmental success. In turn, this has likely led to increased trust in the department's approach which made it easier for the hospital management to approve the request to initiate the project.

We are the only department at the hospital that hasn’t been downsized. In fact, we are given new positions every year. The management sees how we make a difference for the somatically ill children. (Department leader, Oslo University Hospital)

### Appealing Story

The architects and sponsors found the story behind the Outdoor Care Retreat immediately appealing, feeling honored to contribute to a project that could benefit many. This narrative motivated their investment of time and resources. Support from hospital management further underscored the story's appeal, as the department leader noted minimal resistance to the idea at higher organizational levels in the hospital administration. Challenges that arose were typically related to finances and practicalities, rather than the concept itself.

The hospital management and clinic leaders are usually required to be skeptical and consider economy to ensure hospital activity. However, these people were so immediately taken by this idea that we didn’t meet much resistance from that hold. (Department leader, Oslo University Hospital)

### Somewhere Beyond the System

The second theme highlighted the Outdoor Care Retreat's unique design and location as a biophilic space in natural surroundings. Key to this was its placement outside the conventional hospital building, creating a physical distance from conventional spaces. This theme was further divided into (1) healing nature and (2) unique design features.

### Healing Nature

The Outdoor Care Retreat's location in natural surroundings was crucial to its appeal, with all participants noting it as key to the project's success. Contributors found the biophilic design and setting highly attractive. For the architects and key stakeholders, it was important that the design harmonized with the landscape and allowed patients to engage with both indoor and outdoor spaces regardless of their physical limitations. Additionally, the building's proximity to the conventional hospital ensured safety while providing a sense of distance from the hospital experience.

The starting point for the Outdoor Care Retreat was to create a space where children could spend time away from the traumatic experiences they were having and coming close to something natural. Water, sticks, rocks. (Architect)

### Unique Design Features

The final design of the Outdoor Care Retreat closely followed the original architectural sketches, though initial meetings revealed differing opinions among team members. The key stakeholders from Oslo University Hospital focused more on creating a nature-inspired, intimate space reminiscent of a fairytale, while the founder of the Outdoor Care Retreat Foundation emphasized unique architectural features and privacy for hospitalized children and their families. The architects combined these perspectives, using their expertise to meet most of the criteria.

We wanted an outdoorsy and child-friendly design. A place with natural materials that felt fairytale-like and could be used for scavenger hunting and other exciting activities for children. (Frontline employee, Oslo University Hospital)

Discussions about different needs and designs led to a consensus on the final Outdoor Care Retreat design. Team members acknowledged that compromises were necessary to stay within budget while achieving agreement. Key stakeholders prioritized a design appealing to children and reflective of its natural surroundings, along with a dedicated space for psychotherapy and sensitive conversations. While they expressed satisfaction with the outcome, some preferred a softer architectural appearance.

We wanted the space to feel different, perhaps a bit weird. Something people would notice and take a second look at. Thisled to these crooked walls that makes it look like a wooden cottage that a child could have built themselves, something playful, something magical (…) To me, the design is a bit ‘masculine’ and ‘fancy’. At the same time, I realized that a lot of people like that, so maybe it wasn’t such a bad idea. (Frontline employee, Oslo University Hospital)

### Someone Beyond the System

This theme highlights the role of individuals in developing innovative ideas in healthcare design. Participants emphasized that enthusiastic, knowledgeable, and collaborative efforts were crucial to project development. This theme was divided into (1) passion and persuasion, (2) stamina and patience, (3) willingness to take risks, and (4) not a single someone.

### Passion and Persuasion

Key stakeholders consistently reflected on their passion for initiating the project. The founder of the Outdoor Care Retreat Foundation shared how his personal experience as a next-of-kin increased his awareness of the importance of architecture and art in hospitals, promoting his wish for better-designed environments. His enthusiasm resonated with everyone involved in the project. Similarly, the key stakeholders at Oslo University Hospital shared a commitment to transforming healthcare delivery, particularly the frontline employee, who emphasized the benefits of spending time in natural environments outside conventional hospital spaces.

The Outdoor Care Retreat really is a manifestation of [Frontline employee Oslo University Hospital] work. Without her this would never have happened. She is the midwife of the entire project because it is about her story and these meetings with scared children that suddenly opened up when they came outside. (Architect)

Alongside their passion, contributors noted that key stakeholders effectively communicated the project story and potential benefits in a persuasive manner. Sponsors described the team as passionate, trustworthy, experienced, and knowledgeable.

### Stamina and Patience

Key stakeholders noted that the project development was time-consuming and challenging and included bureaucratic challenges, budget constraints, and differing institutional interests. They emphasized that overcoming these obstacles required stamina and patience, driven by a strong belief in the project's benefits for patients and their families. They reflected on the need for both patience and impatience to facilitate project progress, suggesting that the complexities of public healthcare could only be navigated through their persistence.

There were so many meetings. It was surprising to discover how the inner world of public healthcare works. How they think and the time frames they work by. It was a long and truly time-consuming journey. (Founder of the Outdoor Care Retreat Foundation)

### Willingness to Take Risks

Participants discussed the risks associated with initiating and implementing the project, particularly during the early phases when the project's outcome was uncertain and there were no tangible elements to present to potential contributors. According to the project manager from the Hospital of Southern Norway, this was a critical point in the process. He explained how the hospital administration always needs to consider future management of novel services or products, and how this led to worries regarding long-term costs for the hospital in managing the Outdoor Care Retreat. Several participants explained that risk-reducing efforts were made, such as ensuring mutual trust between stakeholders and contributors and transparent communication of financial plans, but that high risk tolerance was still needed for everyone involved with the project, including key stakeholders, the hospital management, the architecture firm, and sponsors.

It's important who you get to work with you on an innovative project. You need people that dare to throw themselves in the water before knowing the temperature. It can be uncomfortable for a while, but it gets better. Unless you freeze to death, of course. (Founder of the Outdoor Care Retreat Foundation)

### Not a Single Someone

The project involved multi-directional collaborations, notably among the hospital, the Outdoor Care Retreat Foundation, and the architecture firm, with sponsors and project managers facilitating these efforts. Participants emphasized the importance of engaging individuals with diverse experiences for project success, highlighting that no single person can provide all the necessary resources for innovation. They particularly noted the contributions of the Outdoor Care Retreat Foundation founder and the frontline employee at Oslo University Hospital, sharing stories that underscored how the project would not have succeeded without their enthusiasm and commitment. The two key stakeholders consistently credited each other for the innovation's success, recounting similar narratives that acknowledged the benefits of their differences. They emphasized how their shared passion for transforming the hospitalization experience for patients and their families enabled them to overcome challenges in a collaborative manner.

Then you have the enthusiasm of [frontline employee Oslo University Hospital). The project wouldn’t have happened if she and the department leader had not wanted it too. The idea needed to be embedded in a department at the hospital. (Founder of the Outdoor Care Retreat Foundation)

The diverse set of professional backgrounds for project success was especially important in ensuring trust and financial transparency. For instance, the project manager at the Hospital of Southern Norway had extensive experience in project management and was able to function as a bridge between the hospital administration and the enthusiastic, but less experienced, project initiators, providing financial advice to key stakeholders while reassuring the hospital administration in their financial concerns.

## Discussion

The present study was designed to provide insights into public healthcare innovation by drawing on experiences from developing the Outdoor Care Retreat. The main findings produced for this study demonstrate how different approaches of moving beyond an established system can help facilitate access to innovative biophilic healthcare design for hospitalized patients. Specifically, the Outdoor Care Retreat project serves as an example of how innovation in public healthcare can flourish due to a combination of people, an organizational culture, and an environmental context that dare challenge common conventions.

One finding is the importance of people with individual and shared abilities and traits that align with necessary traits for innovation success as outlined by previous research (see Thornhill-Miller & Lubart, 2019). In exploring the project from initiation up until implementation, it is apparent that the project was initiated and developed by a group of highly innovative individuals. Key stakeholders all seem to inhabit individual features commonly associated with innovation success, thus supporting existing empirical research and theory in this field (Amabile, 1988; Sternberg & Lubart, 1999; Thornhill-Miller & Lubart, 2019; Zhang & Sternberg, 2011). This is reflected in their intrinsic motivation to break with tradition and try out new ideas, their high tolerance for uncertainty, and their willingness and ability to grow and overcome challenges as the project proceeded. A possible downside to acknowledging individual influences of innovation is expecting too much from people in biophilic design innovation, when other routes to innovation success are possible, such as building an innovative culture at an organizational level.

Indeed, the Outdoor Care Retreat is a unique healthcare innovation that requires an organizational shift from something familiar to something novel. Within this lies the need for an organizational and environmental context that encourages innovation. The Department of Child and Adolescent Mental Health in Hospitals at Oslo University Hospital and their employees are portrayed as a workplace with an organizational culture that encourages novel thoughts and procedures. The creative and holistic approach to healthcare and treatment at the department has led to a culture of doing things differently, nurturing a culture with employees that inhabit similar mindsets and approaches. Additionally, the department leader exhibited traits aligned with transformational leadership previously associated with innovative behavior (Sarooghi et al., 2015), as he motivates and encourages employees to think outside the box. This culture is manifested in every aspect of therapy and treatment, and by extension the Outdoor Care Retreat is considered a physical manifestation of this culture. In addition to the creative culture at department level, the innovation idea was met with enthusiasm and trust at higher levels in the hospital administration. This implies support and trust in the department's approach, thus suggesting that the inclination towards innovative action is evident at several levels within the hospital organization. Thus, the environmental context seemed to play an important role in the project's success and supports previous research on this association (Amabile, 1988; Kennel & Lowndes, 2023; Sarooghi et al., 2015). This insight bridges the need for innovation at both personal and organizational level but underlines the possibility of inspiring an innovation mindset in all people and not simply those who already possess these individual traits. While these are important findings, it is still necessary to acknowledge that the project received external funding by private sponsors to ensure implementation. The hospital remains financially responsible for continued management of the Outdoor Care Retreat but the external funding arguably contributed to innovation success.

Moreover, study findings support the importance of collaboration in multi-disciplinary teams for innovation success. Several participants attributed the successful initiation and development of the project to the diverse set of professional backgrounds, skills, and knowledge in team members. Team members experienced a genuine appreciation of working together with people with a different set of skills and backgrounds, supporting previous examples of beneficial attitudes towards multi-disciplinary teams in healthcare (Sonke et al., 2017). Additionally, findings suggest that not all innovation antecedents at individual level must exist in one individual. Rather, the combination of different skills and individual traits leads to a successful outcome where each individual member contributes where needed whether in terms of personality traits, knowledge, intellectual abilities, motivation, or expertise. These findings resonate with previous literature where a reciprocal and successful collaboration is seen as more beneficial than individual contributions to innovation (Kennel & Lowndes, 2023; Torfing, 2018).

Existing research and literature suggest that humans have an affinity for natural environments and natural materials nature (Kellert, 2018; Kellert & Wilson, 1993). In this study, this assumption is mirrored in the positive experiences, beliefs, and assumptions the participants attach to their meaning of the project's biophilic design and location in natural surroundings. This was especially evident in how the participants with less familiarity with nature and research were immediately taken with the idea of an Outdoor Care Retreat. For the key stakeholders and the architects, the positive association with nature as beneficial for healing could be related to their existing professional knowledge. Still, in general, the present findings correspond with principles of biophilic design and the biophilia hypothesis (Kellert & Wilson, 1993; Wilson, 1984). Thus, the positive beliefs about the benefits of spending time in a natural setting, both inside and outside the Outdoor Care Retreat, likely contributed to the successful development of the project as it quickly gained the attention from the hospital administration and sponsors. This finding might suggest that innovations based on principles of biophilic design are easier to “sell” than more complicated technological innovations in healthcare. While acknowledging the positive attitudes toward the Outdoor Care Retreat design, these attitudes could signal a favorable outlook towards the project in more general terms. That is, making sure that hospitalized children have somewhere to spend time outside the conventional hospital space. This could imply that project success does not hinge on the unique and exclusive architectural design and that implementation could be achieved using a less renowned architectural firm and a simpler design that still considers biophilic principles without being too costly. Nonetheless, the distinctive design and the involvement of a prestigious architectural firm may have facilitated the process of convincing stakeholders in the initial phases of the project.

### Strengths and Limitations

The present study has several strengths. Firstly, it generates knowledge on how to bridge the gap between research from environmental psychology and related fields to the practice of public healthcare using concepts from public innovation research. Exploring concepts from environmental psychology from the perspective of public innovation is uncommon and offers a unique perspective on how research on biophilic design can be integrated and made available to the public. Doing so, the study contributes to both innovation research and research on human-environment connections in a healthcare context. For innovation research, this study supports and adds credibility to established concepts such as the importance of individual and contextual influences on innovation. For environmental psychology, it illustrates how research from this field can be integrated into practice. A related strength is the study's potential to enhance the hospitalization experience for patients (Tracy, 2010). If future healthcare projects draw from the insights of this study, it may lead to greater consideration of the relationship between biophilic design, well-being, and health outcomes. This, in turn, could enhance the hospitalization experience and possibly decrease the need for costly psychological support during and after treatment for patients and their families. A limitation in this study is the possibility of providing an overly favorable view of the Outdoor Care Retreat project as a successful public healthcare innovation. Study participants have all been personally and/or professionally invested in the realization of the project and this could have influenced their narrative of the project's success. Moreover, it is necessary to acknowledge that the authors are familiar with the positive associations between nature, biophilic design, and health outcomes. While not intentional, this perspective may have influenced the analytical process and the themes developed, also leading to a favorable view of the Outdoor Care Retreat.

### Conclusion and Future Directions

The present study provides valuable insights into the development of biophilic design alternatives in public healthcare in the current and similar contexts. Although the case study is unique, it highlights how implementation of biophilic design can benefit from innovation at individual, organizational, and environmental levels. The study mainly explores innovation success and future studies should build on present findings to explore barriers for biophilic design innovation as well as more thorough exploration into cost benefit analysis for the present case study and similar projects. Furthermore, emphasis should be placed on the use and user satisfaction of these kinds of projects, preferably with a quantitative methodology as this perspective is currently lacking. In general, the research area could be advanced by focusing on each distinct project phase in the present or similar case studies to provide specific knowledge of essential milestones and related drivers. This will provide a solid framework for further research on innovative biophilic design in public healthcare and can ensure increased access to research-based environments that could improve the hospitalization experience for patients and reduce short-term and long-term negative consequences associated with illness and hospitalization.

## Implications for Practice

- Apply narrative strategies to build support for biophilic design in healthcare. Findings indicate that designers can benefit from a compelling project story that can facilitate stakeholder engagement and resource allocation. Designers should frame biophilic elements within patient-centered narratives that emphasize therapeutic benefits, aligning with principles of research-based design.
- Ensure partnerships with visionary stakeholders in public healthcare to challenge conventional healthcare design norms. Designers can advance biophilic innovation by identifying and collaborating with stakeholders who advocate for transformative, human-centered environments.
- Contextualize biophilic design interventions to align with both organizational culture and local ecological conditions. Designers should conduct early-stage contextual assessments and engage interdisciplinary teams to ensure biophilic features that are ecologically appropriate and operationally feasible.
